# Microbial Dysbiosis During Simian Immunodeficiency Virus Infection is Partially Reverted with Combination Anti-retroviral Therapy

**DOI:** 10.1038/s41598-020-63196-0

**Published:** 2020-04-14

**Authors:** Faith C. Blum, Britney L. Hardy, Kimberly A. Bishop-Lilly, Kenneth G. Frey, Theron Hamilton, James B. Whitney, Mark G. Lewis, D. Scott Merrell, Joseph J. Mattapallil

**Affiliations:** 1 F. Edward Hébert School of Medicine, Uniformed Services University, Bethesda, MD United States; 2 Genomics & Bioinformatics Department, Naval Medical Research Center, Biological Defense Research Directorate, Fort Detrick, MD United States; 3 Center for Virology and Vaccine Research, Beth Israel Deaconess Medical Center, Harvard Medical School, Boston, MA United States; 4 Ragon Institute of MGH, MIT, and Harvard, Cambridge, MA United States; 5 Bioqual, Rockville, MD United States

**Keywords:** Microbiome, Viral infection

## Abstract

Human immunodeficiency virus (HIV) infection is characterized by a massive loss of CD4 T cells in the gastrointestinal tract (GIT) that is accompanied by changes in the gut microbiome and microbial translocation that contribute to inflammation and chronic immune activation. Though highly active antiretroviral therapy (HAART) has led to better long-term outcomes in HIV infected patients, it has not been as effective at reverting pathogenesis in the GIT. Using the simian immunodeficiency virus (SIV) infection model, we show that combination antiretroviral therapy (c-ART) partially reverted microbial dysbiosis observed during SIV infection. Though the relative abundance of bacteria, their richness or diversity did not significantly differ between infected and treated animals, microbial dysbiosis was evident via multiple beta diversity metrics: Jaccard similarity coefficient, Bray-Curtis similarity coefficient, and Yue & Clayton theta similarity coefficient. Principal coordinates analysis (PCoA) clustered SIV-infected untreated animals away from healthy and treated animals that were clustered closely, indicating that c-ART partially reversed the gut dysbiosis associated with SIV infection. Metastats analysis identified specific operational taxonomic units (OTUs) falling within the *Streptococcus*, *Prevotella*, *Acinetobacter*, *Treponema*, and *Lactobacillus* genera that were differentially represented across the three groups. Our results suggest that complete viral suppression with c-ART could potentially revert microbial dysbiosis observed during SIV and HIV infections.

## Introduction

The gastrointestinal tract (GIT) is colonized by microbes that contribute to its development and maintenance. However, numerous disease states have been shown to alter the composition of the gut microbiota. This dysbiosis is often accompanied by translocation of microbes or their products across the mucosal epithelium, which in turn exacerbates inflammatory conditions in the gut mucosa.

Human immunodeficiency virus (HIV) infection is characterized by dramatic alterations in the gut microenvironment during the early stages of infection that is accompanied by significant levels of viral replication, CD4 T cell depletion, compromise of epithelial barrier integrity, and altered immune homeostasis . Loss of barrier integrity leads to microbial translocation that, in turn, contributes to immune activation and disease progression^1^. Interestingly, a number of studies have documented changes in the composition of the gut microbiota during progressive HIV infection as compared to healthy individuals^2–4^; these changes often include a loss of *Bacteroides* and an enrichment of *Proteobacteria*^5–7^. Dillion *et al*.^5^ showed that HIV infected patients have a higher abundance of *Proteobacteria* and a decreased prevalence of *Firmicutes* when compared to healthy subjects. Others have reported decreased microbial richness^8^ and significant alterations in *Prevotella* during chronic HIV infection^7,9–12^. A decrease in the abundance of butyrate producing bacteria was found to correlate with microbial translocation and immune activation in HIV infected subjects^13^.

Apparent changes in the gut microbiota during simian immunodeficiency virus (SIV) infection are less congruent. While rhesus macaques progressing to AIDS were found to display an expansion of enteropathogens^14^, others have reported that the overall microbiota composition was not dramatically altered in SIV infected animals as compared to uninfected animals^14–17^. Interestingly, a subset of SIV infected macaques with severe illness exhibited altered bacterial β-diversity with an increase in the abundance of *Enterobacteriaceae* and *Moraxellaceae*; these changes were similar to those of HIV infected subjects with low CD4 T cell counts and had an increased prevalence of bacterial enteropathogens in their fecal samples^18^. Glavan *et al*.^19^ on the other hand reported that decreased expression of pathogen recognition receptors in the gut mucosa during the acute stages of SIV infection correlated with an increased abundance of numerous taxa of pathogenic bacteria.

The advent of Highly Active Anti-Retroviral Therapy (HAART) has led to better long-term outcomes for HIV infected patients. However, numerous studies have documented persistence of immune activation during HAART^20–22^. Given the association of translocated microbial products with chronic immune activation, there has been a significant interest in understanding the effects of HAART on the gut microbiota. Though the composition of the microbiota did not significantly differ from that of untreated HIV infected subjects, initiation of HAART was associated with altered gut dysbiosis^6,9^. Interestingly, Mutlu *et al*.^6^ reported that HIV infected patients under HAART displayed a loss of commensal taxa and a gain of some pathogenic bacterial taxa. Of note, lower microbial richness and diversity has been associated with poor CD4 T cell reconstitution in HIV infected subjects under HAART^23,24^.

In terms of SIV models, SIV infected pigtail macaques were found to have lower relative amounts of *Proteobacteria* without major changes in either *Bacteriodetes* or *Firmicutes*^25^. Anti-retroviral therapy was accompanied by a significant decrease in the relative amounts of *Firmicutes* and a concomitant increase in *Proteobacteria*. These changes were apparent even though the relative amounts of *Proteobacteria* did not significantly differ from the first 20 days of SIV infection, suggesting that ART likely restores some of the dysbiotic bacteria. It is not clear if similar changes occur in SIV infecetd rhesus macaques .

To address this question, we examined the fecal microbiome of rhesus macaques that were infected with SIVmac251 for 9 weeks and compared them to healthy (uninfected) and c-ART treated animals. Rhesus macaques have been widely used as a model to study HIV pathogenesis and anti-retroviral therapy^26–61^. Our results showed that even though the relative abundance of bacteria or their richness, and diversity did not significantly differ between infected and treated animals, microbial dysbiosis was evident during SIV infection via multiple beta diversity calculators : Jaccard index, Bray-Curtis index, and Yue & Clayton theta coefficient. Furthermore, when visualized by principle coordinates analysis (PCoA), SIV infected untreated animals clustered separately from the healthy and c-ART treated animals, suggesting that c-ART partially reversed the dysbiosis that was apparent during SIV infection.

## Results

### Study population

**Figure 1:**
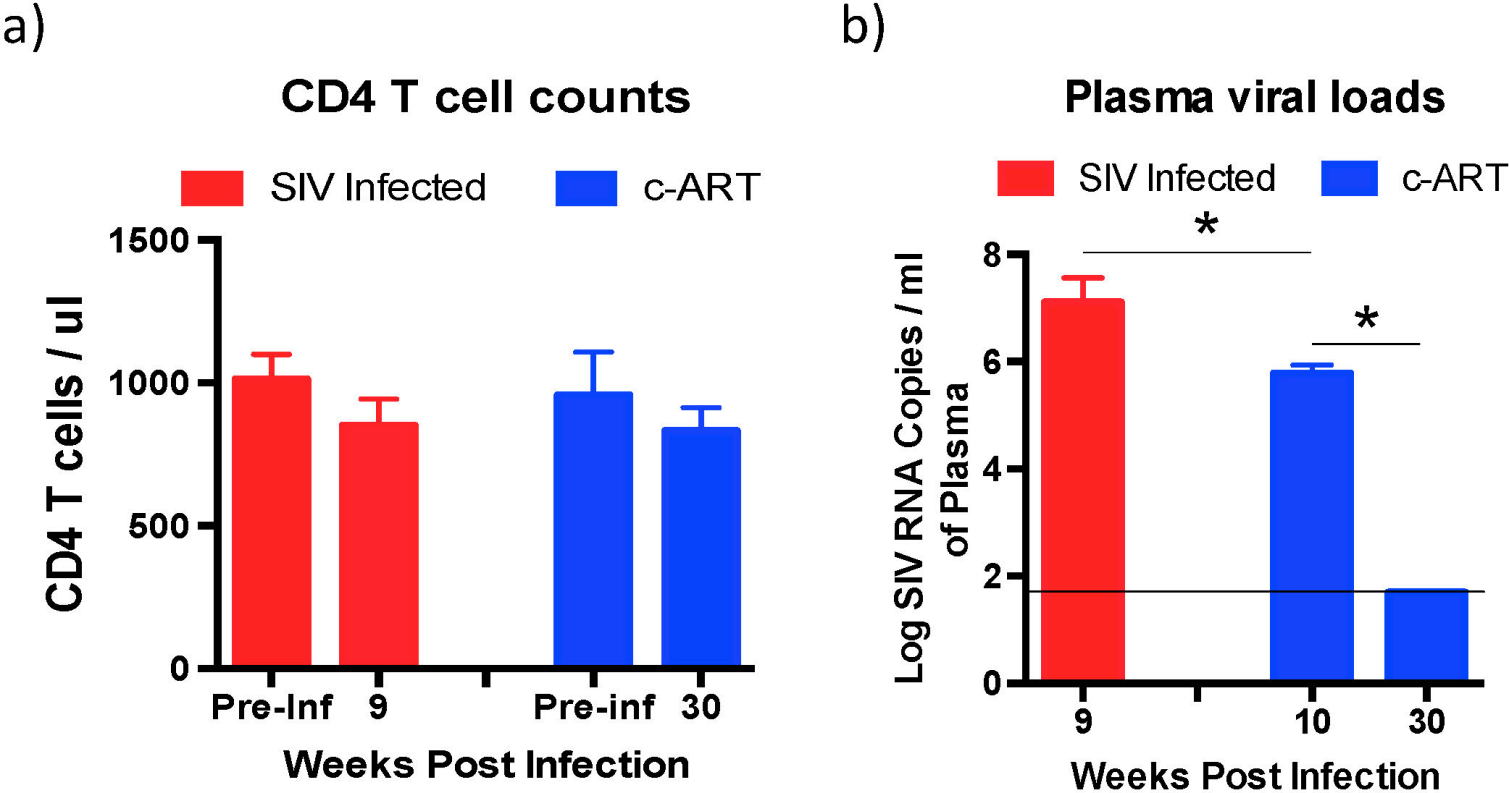
Absolute CD4 T cell counts and plasma viral loads. (**a**) Peripheral blood CD4 T cell counts were determined at 9 weeks PI from the SIV infected untreated animals and at 30 weeks PI from the SIV infected c-ART treated animals and compared to each animals pre-infection values. (**b**) Plasma viral loads in SIV infected untreated and SIV infected c-ART treated animals. Plasma viral loads were determined at 9 weeks PI from the SIV infected untreated animals and at 10 weeks PI prior to initiation of c-ART, and at 30 weeks PI after 20 weeks of continuous c-ART from the SIV infected c-ART treated animals.

Fecal samples that were collected from a cross-section of healthy (n = 7), SIV infected untreated (n = 6), and SIV infected c-ART treated (n = 10) rhesus macaques (~2.5–4 years old; males) were used in this study. All the animals were housed at Bioqual and received similar diets. Absolute CD4 T cell counts were determined at 9 weeks post-infection (PI) in the SIV infected untreated group of animals and at 30 weeks PI in the c-ART treated group of animals and compared to their pre-infection CD4 T cells counts. There was no significant difference in the CD4 T cell counts between the SIV and c-ART group of animals relative to their pre-infection values (Fig. 1a).

To determine if the levels of plasma viremia were similar between the SIV infected untreated and c-ART treated group of animals prior to initiating therapy, we compared the plasma viral loads from SIV infected untreated group of animals at 9 weeks PI to c-ART treated group of animals at 10 weeks PI when c-ART was initiated. There was a significant difference in the plasma viral loads (Fig. 1b) between the SIV infected group of animals (9 weeks post-SIV infection; ~5 ×10^7^ copies of SIV RNA/ ml of plasma) as compared to the c-ART group of animals at the time of initiation of c-ART (10 weeks post-SIV infection; ~6 ×10^5^ copies of SIV RNA/ ml of plasma). To examine if c-ART was effective at suppressing viremia we determined plasma viral loads at 30 weeks PI (20 weeks after c-ART initiation) and compared them to plasma viral loads at the time of c-ART initiation at 10 weeks PI. Continuous c-ART significantly suppressed plasma viral loads at 30 weeks PI to levels that were below the limits of detection (<50 copies/ ml of plasma).

### rRNA gene sequencing

Earlier studies had reported gut dysbiosis during progressive SIV infection^14,18^. To determine if SIV infection was accompanied by microbial dysbiosis, we examined the fecal microbiota from SIV infected untreated macaques by sequencing the V4 region of the 16S rRNA gene and compared them to that of c-ART treated and healthy group of animals.

A total of 7,819,760 raw reads were obtained, of which 4,779,359 reads remained after quality filtering and sequence processing. The samples contained an average of 207,798 sequences (range of 108,414 to 308,433). These sequences clustered into a total of 27,657 operational taxonomic units (OTUs), with an average of 2,997 OTUs per sample (range: 2,395–3,756) when classified using the RDP collection. The raw sequencing reads can be accessed using the National Center for Biotechnology Information (NCBI) Bioproject ID# PRJNA561197 (http://www.ncbi.nlm.nih.gov/bioproject/561197).

### Microbiota composition, richness, and diversity

**Figure 2:**
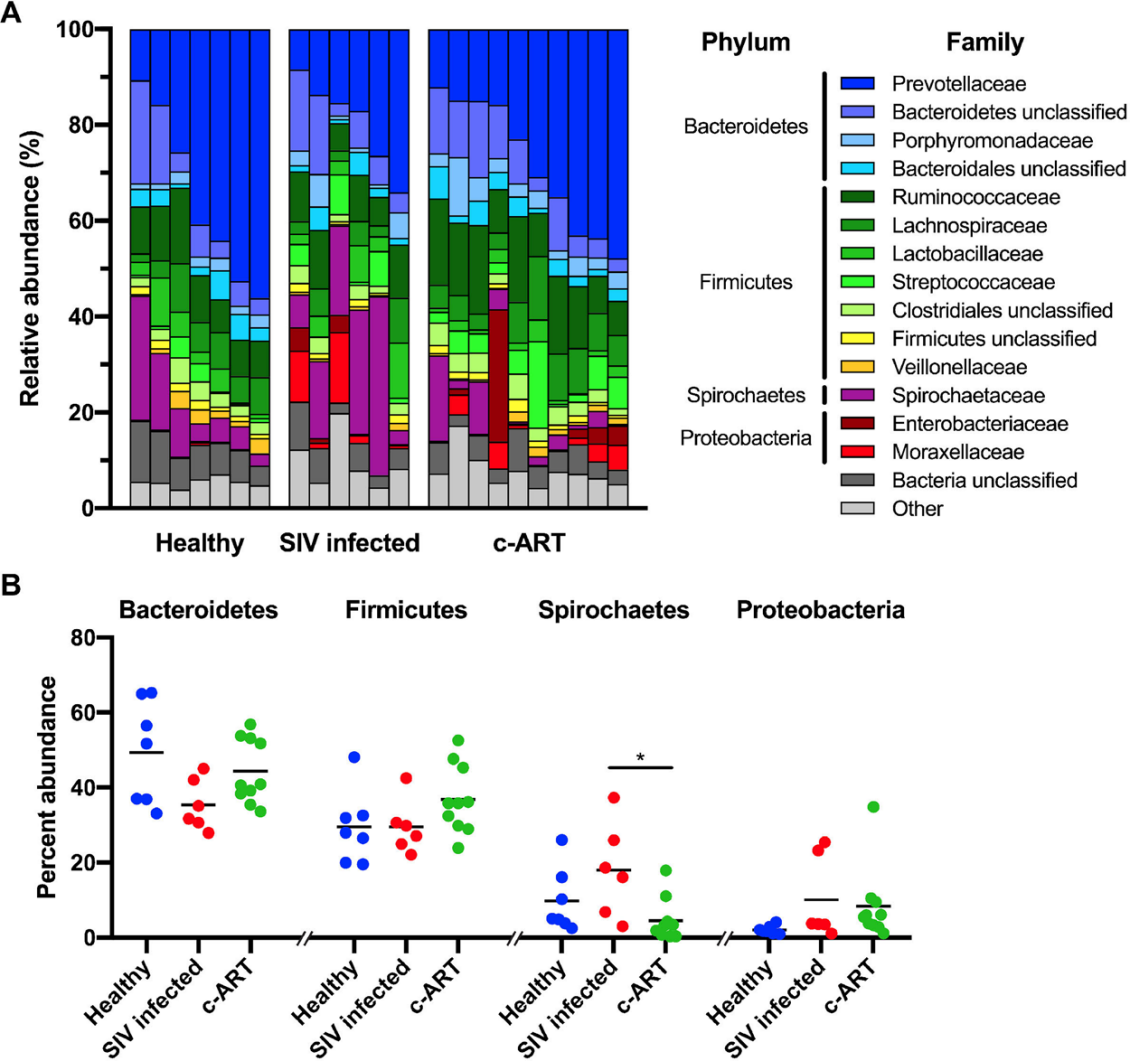
Changes in rhesus macaque gastrointestinal microbiota during SIV infection and c-ART. (**A**) Relative abundance of family-level OTUs, with those families constituting ≥1% of the total sequences shown. “Other” comprises all remaining sequences. OTUs were classified to the Ribosomal Database Project (RDP) collection based on V4 16S rRNA gene sequencing from fecal material. (**B**) Percent abundance of phylum-level OTUs, with those phyla constituting ≥1% of the total sequences shown. Each symbol represents one sample, with the mean shown as a solid black line. Within each phyla, differences in abundance between the treatment groups was tested by one-way ANOVA with Tukey’s test for multiple comparisons, where *P* < 0.05 = *.

The relative abundance of the bacterial families that represented >1% of the total sequences are shown in Fig. 2A, and the relative abundance data for all samples are included in Suppl. Table 1. In line with earlier studies^17,25,62^, the major phyla present were *Bacteroidetes* (44%, average percentage across all samples), *Firmicutes* (33%), and *Spirochaetes* (10%), with a minor contribution from *Proteobacteria* (7%). Almost half of the total sequences classified to three families: *Prevotellaceae* (27%, member of the *Bacteroidetes*), *Ruminococaceae* (11%, member of the *Firmicutes*), and *Spirochaetaceae* (10%, member of the *Spirochaetes*). Visually, the overall composition of the microbiota did not appear to dramatically differ between the healthy, SIV infected untreated, and c-ART treated groups (Fig. 2A). The one exception was the increase in the abundance of *Spirochaetes* found in the SIV infected group of animals (Fig. 2B).

To deal with differences in sequencing depth across individual samples, the sequences were randomly subsampled to the lowest number of sequences found in a sample (108,414) prior to diversity analyses. The Good’s coverage of the subsampled dataset averaged 99.0% (range: 98.4%-99.2%) and rarefaction analysis (Suppl. Fig. 1) suggested that subsampled sequences still accurately represented the overall number of OTUs present in a sample. This is in line with previous observations that the richness of bacterial taxa at this site is very high^17^. However, the slopes of the rarefaction curves suggest that even with sequencing depth of >100,000 sequences per sample, some rare bacteria within the rhesus macaque gut microbiota would still not be detected.

**Figure 3:**
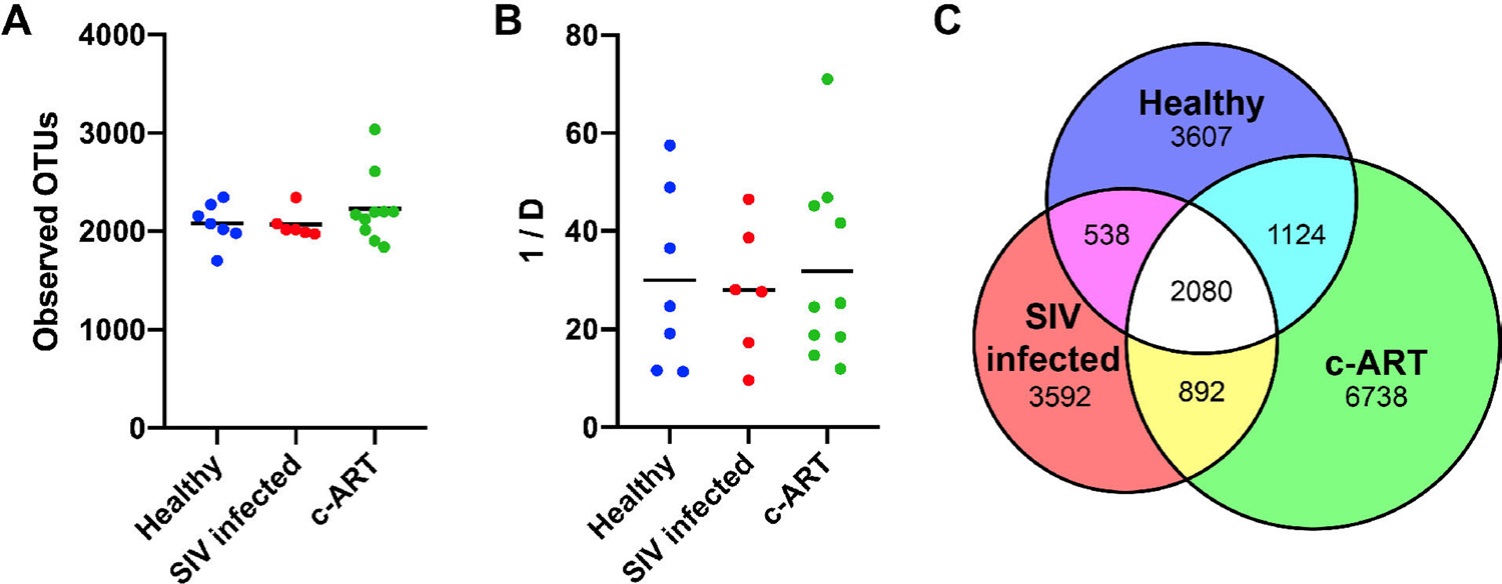
Alpha diversity of treatment groups. (**A**) The bacterial richness of each sample, as calculated by the number of observed OTUs. (**B**) The diversity of each sample as calculated by the inverse Simpson (1/D) diversity calculator, such that the higher the number, the greater the diversity. In (**A,B**), each symbol represents one sample, with the mean shown as a solid line. No groups were statistically different, as tested by a one-way ANOVA with Tukey’s multiple comparison. (**C**) Venn diagram showing the number of OTUs shared between each group. All calculations were performed after subsampling.

To measure bacterial richness, the number of observed OTUs per sample and group were determined using the subsampled data (Fig. 3A). Overall, the bacterial richness of each group was not different from the other groups, with an average number of 2078 OTUs from the healthy group, 2068 from the SIV-infected group, and 2228 from the c-ART treated group (*P* > 0.05, using a one-way ANOVA with Tukey’s multiple comparison test) of animals. Similarly, overall diversity analysis using the inverse Simpson index (invsimpson, Fig. 3B) revealed that the groups were not different (*P* > 0.05, using a one-way ANOVA with Tukey’s multiple comparisons test); diversity values for the healthy (29.97), SIV infected (27.94), and c-ART treated (31.85) samples were similar.

To assess shared richness between the three groups, the number of shared and unshared OTUs was determined (Fig. 3C). Of the 18,571 observed OTUs in the subsampled dataset, 2080 were shared between all groups. Additionally, while each individual group shared a roughly equal proportion of OTUs with the other individual groups, the healthy and c-ART treated groups shared a slightly higher number of OTUs with one another as compared to the SIV infected untreated group. Furthermore, the c-ART treated group contained a much larger number of unique OTUs as compared to the other groups; the healthy and SIV infected untreated groups each contained approximately 3600 unique OTUs, whereas the c-ART treated group contained 6700 unique OTUs. Taken together, these data suggest that while dramatic dysbiosis was not readily apparent (Fig. 2), the composition of the fecal microbiota differed between groups.

**Figure 4:**
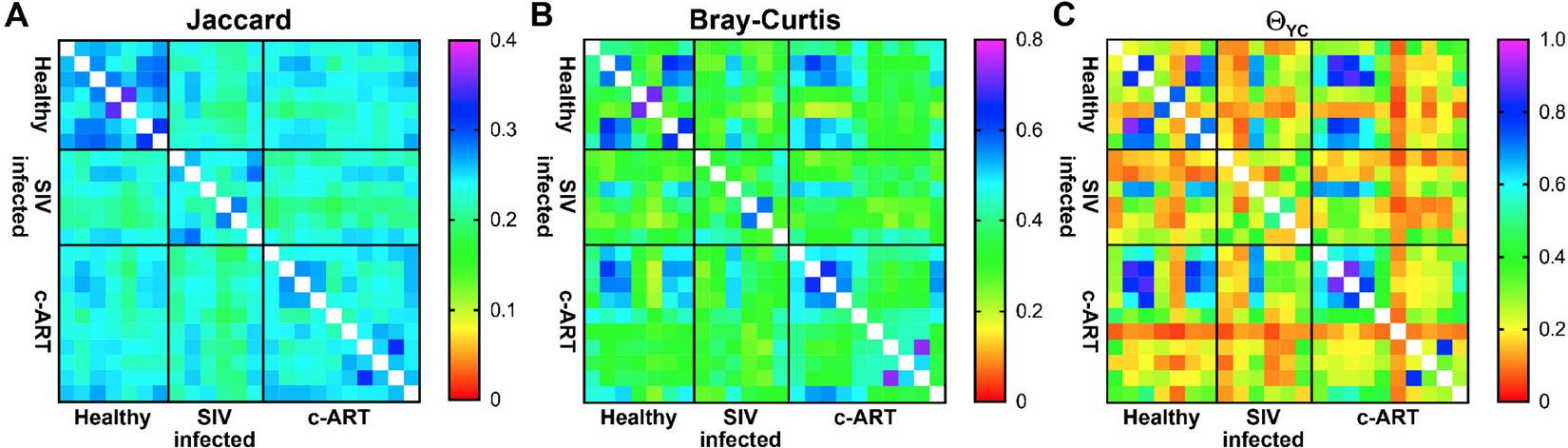
Heat map visualization of beta-diversity calculators. Beta-diversity analysis was performed on subsampled data using the Jaccard (**A**), Bray-Curtis (**B**), and Yue & Clayton theta (Θ_YC_) coefficient (**C**) calculators. The output of all three calculators ranges from 0–1. Similarity is plotted; thus, 0 is dissimilar, and 1 is similar. The color scale for each calculator is shown adjacent to each heat map.

**Figure 5:**
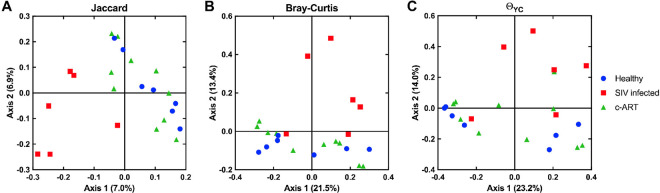
Principal coordinates analysis (PCoA) visualization of beta-diversity calculators. Beta-diversity analysis was performed on subsampled data using the Jaccard (**A**), Bray-Curtis **(B**), and Yue & Clayton theta (Θ_YC_) coefficient (**C**) calculators. Each symbol represents one sample. The variation described by axis 1 and axis 2 is shown in parentheses.

To more thoroughly assess the differences between the groups, we performed beta-diversity analyses using multiple distance calculators: the Jaccard index^63^, which is calculated based on the membership of the community, the Bray-Curtis index^64^, which is calculated based on the structure of the community, and the Yue & Clayton theta (Θ_YC_) coefficient^65^, which is also calculated based on community structure, but additionally takes into account the relative abundance of each OTU. The resulting distance matrices were visualized as heat maps (Fig. 4) and were also subjected to Principal Coordinates Analysis (PCoA) as an additional way to visualize the results of the distance calculators (Fig. 5). The PCoA plots generated from all three distance metrics revealed that the SIV infected untreated group clustered away from the healthy and c-ART treated groups, which appeared similar to each other (Fig. 5).

To determine whether these differences in diversity were statistically significant, analysis by molecular variance (AMOVA) was performed for each distance matrix (Table 1). All comparisons were significant (*P* < 0.05) with the exception of the Θ_YC_ coefficient between healthy vs. c-ART treated groups (*P* = 0.136). This suggests that when community membership is solely considered, as with the Jaccard index, the fecal microbiota of healthy and c-ART treated rhesus macaques were different. Similarly, the Bray-Curtis index, a measure of community structure, found that the two groups were different. However, when the structure of the communities as calculated by Θ_YC_ coefficient was considered, the healthy and c-ART treated rhesus macaques were not different. One distinct difference between the two calculators is that the Bray-Curtis index is calculated using the raw abundance in each OTU, whereas the Θ_YC_ coefficient is calculated using the relative abundance of each OTU. Given the differences in results and statistics when beta-diversity was calculated, this distinction appears to be very important for this dataset. Thus, even though differences exist between all three treatment groups, SIV infection appears to result in changes in beta diversity that are at least partially restored by c-ART.

**Table 1:** Analysis by molecular variance (AMOVA) values for treatment group differences in beta diversity.

Comparison groups	Jaccard	Bray-Curtis	Θ_YC_
All groups	**<0.001**	**<0.001**	**0.008**
Healthy vs. SIV-infected	**<0.001**	**0.006**	**0.007**
SIV-infected vs. c-ART treated	**<0.001**	**0.003**	**0.015**
Healthy vs. c-ART treated	**0.001**	**0.030**	0.136

^*^Significant *P* values are shown in bold.

### OTU specific changes

Finally, to determine which specific OTUs were differentially abundant between the groups, Metastats analysis^66^ was performed on OTUs whose average abundance was ≥1% in at least one group. A total of 19 OTUs were identified as significantly different (*P* < 0.05) between the three groups (Table 2 and Suppl. Fig. 2). If we expanded the analysis to consider OTUs whose average abundance was ≥0.1% in at least one group, 70 additional OTUs were found to be significantly different (Suppl. Table 2). As these analyses were performed on randomly subsampled data, to allow repeatability of the analysis, the relative abundance data for all subsampled samples are included in Suppl. Table 3. Of the 19 OTUs identified in the ≥1% abundance category, 8 were different between healthy and SIV infected untreated, 7 were different between SIV infected untreated and c-ART treated, and 12 were different between healthy and c-ART treated groups. Classification with the RDB reference database revealed that the OTUs that differed included members of the *Streptococcus*, *Prevotella*, *Acinetobacter*, *Treponema*, and *Lactobacillus* genera.

**Table 2:** Metastats analysis.

OTU	Classification^a^		Mean abundance (%)			Metastats *P* value		
	According to RDP database	According to Greengenes database	Healthy	SIV infected	c-ART treated	Healthy vs SIV infected	SIV infected vs c-ART treated	Healthy vs c-ART treated
OTU2	*Streptococcus*	*Streptococcus luteciae*	1.44	3.49	4.95	0.215	0.620	**0.034**
OTU3	*Porphyromonadaceae* (family)	*S24-7* (family)	1.60	2.12	3.53	0.707	0.379	**0.048**
OTU4	*Prevotella*	*Prevotella copri*	3.82	0.40	1.91	**0.001**	**0.002**	0.071
OTU6	*Acinetobacter*	*Acinetobacter*	0.10	4.65	1.66	0.059	0.286	**0.008**
OTU7	*Treponema*	*Treponema*	0.71	7.51	0.14	0.068	0.052	**0.008**
OTU8	*Prevotella*	*Prevotella copri*	2.29	0.50	2.51	**0.007**	**0.002**	0.852
OTU10	*Treponema*	*Treponema*	0.43	3.98	1.06	0.076	0.178	**0.042**
OTU11	*Prevotella*	*Prevotella copri*	2.00	0.05	2.61	**0.040**	**0.013**	0.753
OTU13	*Treponema*	*Treponema*	2.04	0.06	1.68	**0.044**	0.086	0.848
OTU14	*Bacteroidetes* (phylum)	*p-2534-18B5* (family)	0.48	1.04	2.14	0.335	0.215	**0.017**
OTU15	*Prevotellaceae* (family)	*[Prevotella]*	1.36	0.63	1.70	0.282	**0.037**	0.723
OTU19	*Lactobacillus*	*Lactobacillus*	1.16	1.62	0.22	0.668	**0.007**	0.106
OTU23	*Treponema*	*Treponema*	0.70	2.68	0.08	0.259	0.108	**0.038**
OTU25	*Dialister*	*Dialister*	1.42	0.40	0.73	**0.012**	0.122	0.110
OTU27	*Bacteroidales* (order)	*YRC22*	1.81	0.36	0.33	**0.041**	0.899	**0.036**
OTU33	*Enterobacteriaceae* (family)	*Enterobacteriaceae* (family)	0.05	1.19	0.79	0.096	0.728	**0.030**
OTU37	*Bacteroidetes* (phylum)	*Bacteroidetes* (phylum)	0.15	0.27	1.04	0.476	**0.020**	**0.005**
OTU42	*Bacteria* (kingdom)	*Treponema*	1.10	0.13	0.04	**0.010**	0.629	**0.004**
OTU54	*Lactobacillus*	*Lactobacillus*	0.17	1.07	0.02	**0.031**	**0.013**	**0.017**

OTU number is listed, together with the classification according to the Ribosomal Database Project (RDP) and the Greengenes databases. OTUs constituting ≥1% mean abundance for any group (healthy, SIV infected, or c-ART treated) are shown. Analysis was performed from a subsampled dataset. Metastats P values < 0.05 are shown in bold. ^a^Genus and species are italicized. If the OTU was not classified to genus, the taxonomic group to which it classified is listed, with that taxonomic level in parantheses. No species information was available for OTUs classified to the RDP collection.

**Figure 6:**
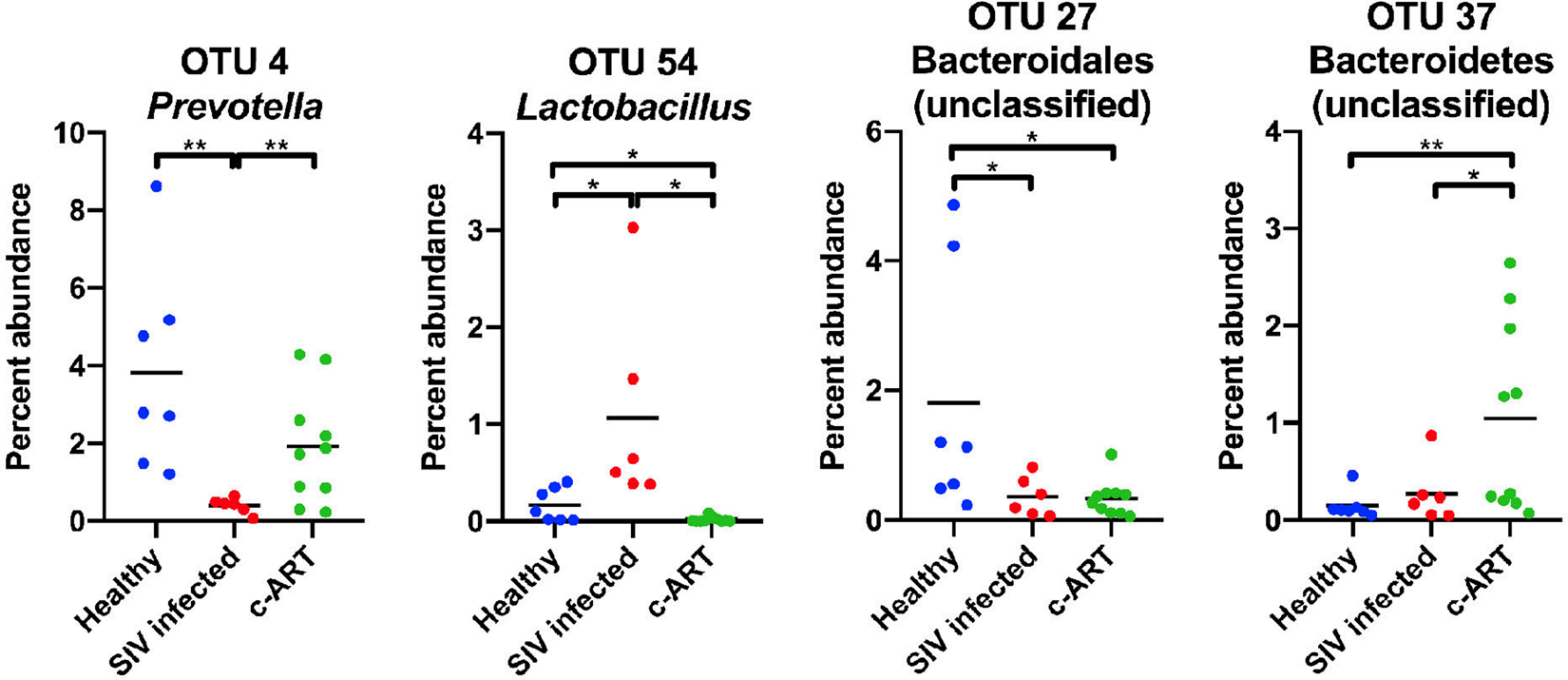
Scatter plots of representative OTUs identified as statistically different by Metastats. Percent abundance of the OTU is plotted, and each symbol represents one sample, with the mean shown as a solid line. OTUs decreased (OTU4 and OTU27) or increased (OTU54 and OTU37) in mean abundance from healthy to SIV infected, and subsequently increased (OTU4 and OTU37) or decreased (OTU27 and OTU54) from SIV infected to c-ART treated groups. Calculations were performed from a subsampled dataset. Statistical significance was tested by the mothur implementation of Metastats, with *P* < 0.05 = *, and *P* < 0.01 = **.

To confirm and extend the classification to the species level where possible, the OTUs were also classified against the Greengenes database. Using this approach OTU2 was classified as *Streptococcus luteciae*, and several other OTUs were classified as *Prevotella copri*. For example, OTU4, OTU8, and OTU11 each classified as *Prevotella copri*; these OTUs were less abundant in the SIV infected group of animals as compared to both healthy and c-ART treated group of animals (Fig. 6). Thus, *Prevotella copri* appears to be a member of the microbiota of healthy rhesus macaques that is reduced over the course of SIV infection, and is restored after c-ART.

OTU15, a member of the family *Prevotellaceae*, followed a similar pattern, though the difference between the healthy and SIV infected untreated groups did not achieve statistical significance (Table 2). In contrast, several OTUs showed an increase or decrease in abundance when healthy animals were compared with SIV infected untreated animals, but this trend was not reversed in c-ART treated animals; these include OTU2 (*Streptococcus luteciae*), OTU3 (family S24-7), OTU14 (family p-2534-18B5), OTU27 (*YRC22*), OTU33 (family *Enterobacteriaceae*), OTU37 (phylum *Bacteroidetes*), and OTU42 (*Treponema*). Thus, even though c-ART appears to shift the fecal microbiota to be more similar to healthy animals (Fig. 5 and Table 1), ultimately treatment was unable to completely alleviate all SIV-associated dysbiosis within the timeframe examined in these animals.

## Discussion

HIV and SIV infections are accompanied by chronic immune activation and disease progression that is likely aided by the translocation of microbial products across the gut epithelium. Though gut dysbiosis has been well documented during HIV infection, whether similar changes occur during SIV infection is less clear. SIV infected pigtailed macaques have been reported to display altered gut microbiota^25^, whereas these changes were not as readily apparent in SIV infected rhesus macaques^67^. At a broader level, our results are in line with these findings as the overall microbiota composition did not seem to differ between the SIV infected untreated and other groups of macaques. However, dysbiosis was apparent when the beta-diversity was examined using multiple metrics, suggesting that while infection-associated changes in the composition of gut microbiota are subtle, they were apparent during SIV infection.

Interestingly, animals under c-ART for a period of 20 weeks (30 weeks PI) clustered with healthy animals (Fig. 5). These animals initiated c-ART at about the same time (10 weeks PI) that the SIV infected untreated group of animals were sampled at 9 weeks PI. Unfortunately, we did not have access to fecal samples from the c-ART group of animals at the time of c-ART initiation (10 weeks PI) to directly compare these time points. However, the cross-sectional comparisons between the 3 groups of animals and the clustering of the SIV infected untreated group away from the c-ART treated and healthy animals (Fig. 5) suggest that c-ART partially reverts the dysbiosis observed during SIV infection.

It is important to consider our results within the context of prior studies. Earlier studies have reported that SIV infection *per se* did not alter gut microbiota in rhesus macaques^67^. However, Dillon *et al*.^5^ reported an increase in the percent abundance of the *Prevotella* genus in HIV-infected individuals. This is in contrast to the decreased abundance of specific *Prevotella* OTUs that we observed in the SIV infected untreated group of animals (Table 2). Mutlu *et al*.^6^ reported several taxa that were indicator species of healthy human subjects as compared to HIV infected patients: these included *Dialister* in the control group and *Enterobactericeae* and *Prevotella* in the HIV group. Similarly, we observed a higher abundance of OTU25 (*Dialister*) in the healthy as compared to SIV infected untreated group, and a lower abundance of OTU33 (*Enterobacteriaceae*) in the healthy group as compared to the c-ART treated group. However, unlike the prior study^6^, we observed several OTUs of *Prevotella* that were higher in the healthy and c-ART treated groups as compared to the SIV infected untreated group of animals (OTUs 4, 8, and 11). The percent abundance of *Spirochaetes* was significantly higher in the SIV infected untreated group as compared to the c-ART treated group (Fig. 2B), though it did not differ from that of healthy animals, suggesting that c-ART likely leads to a reduction in the abundance of *Spirochaetes*. As has been reported in SIV infected pigtail macaques, there was no major difference in the relative abundance of either *Bacteriodetes* or *Firmicutes* between the groups^25^.

There are a number of factors such as the experimental design, variation in animals sampled, their diets, etc., that may contribute to the differences between the various studies that have examined changes in the gut microbiota during HIV and SIV infections. In our study, it is also likely that the higher viremia in the SIV infected untreated group (~7 log of SIV/ ml of plasma at 9 weeks PI) as compared to the c-ART treated group (~ 6 logs of SIV/ ml of plasma at 10 weeks PI) at the time of initiation of therapy could have played a role. There was, however, no major difference in the peripheral blood CD4 T cell counts between the SIV infected untreated and c-ART treated animals. It is also important to point out that the described study was cross-sectional in nature; clearly longitudinal sampling from the same animal over time would yield better insights into the animal-specific changes in gut microbiota during the course of SIV infection.

In conclusion, our results show that SIV infection differentially alters the composition of the gut microbiota and that this change is not readily apparent in animals receiving c-ART. This finding, combined with the clustering of c-ART-treated animals with healthy macaques, strongly suggests that highly suppressive ART likely reverts some of the dysbiosis associated with SIV infection.

## Materials and Methods

### Animals and fecal samples

Fecal samples were collected without handling the animals from ~2.5 to 4 year old male rhesus macaques (*Macaca mulatta*) of Indian origin as follows: healthy (n = 7), SIV infected untreated (n = 6), and SIV infected c-ART treated (n = 10) animals. Sixteen animals were infected with 100 animal infectious doses of SIVmac251 intravenously. The c-ART group of animals was treated with a combination of PMPA (Tenofovir; 30 mg/ Kg BW/ daily), FTC (Emtricitabine; 20 mg/ Kg BW/ daily), and DTG (Doultegravir; 2.5 mg/ Kg BW/ daily) subcutaneously for a period of 20 weeks starting at 10 weeks PI.

All animals were housed at Bioqual in accordance with the recommendations of the Association for Assessment and Accreditation of Laboratory Animal Care International Standards and NIH Guide for the Care and Use of Laboratory Animals of the United States, and were a part of protocols that were previously approved by the Institutional Animal Use and Care Committee (IACUC) of BIOQUAL (protocol# 17-030). As the fecal samples were collected without handling the animals, a separate IACUC protocol was not required by the IACUC at Bioqual or USUHS. All the animals were seronegative for SIV, simian retrovirus and simian T-cell leukemia virus type-1 prior to being enrolled in these studies. Fecal samples were collected, immediately snap frozen in liquid nitrogen, and transported to the laboratory for analysis.

### Bacterial DNA extraction

Approximately 200–300 mg of fecal material from each animal was used for DNA isolation. Total bacterial genomic DNA was extracted using the PowerSoil-htp 96 Well Soil DNA Isolation Kit (MOBIO). PCR amplification and sequencing protocols were adapted from the Earth Microbiome Project (http://press.igsb.anl.gov/earthmicrobiome/protocols-and-standards/16s/). Briefly, the V4 region of the bacterial 16S rRNA gene was PCR-amplified using barcoded primers to generate paired-end reads^68^. Each 25-µL PCR mixture contained 10 µL 5 PRIME HotMasterMix (QuantaBio), 0.5 µL of each 10 µM primer, 1 µL extracted genomic DNA, and 13 µL sterile water. PCR amplification was performed using the following settings: 94 °C for 3 min; followed by 35 cycles of 94 °C for 45 s, 50 °C for 1 min, and 72 °C for 90 s; followed by 72 °C for 10 min. Each sample was amplified in triplicate and pooled, resulting in 75 µL of PCR product. Products were visualized by gel electrophoresis and the concentration of PCR-amplified DNA fragments was determined using a Nanodrop (Thermo). About 240 ng of each amplicon was next pooled and the pooled amplicons were cleaned using the UltraClean PCR Clean-Up Kit (MOBIO). Samples were assessed using a Bioanalyzer (Agilent Technologies) for quality and average size distribution and via Qubit (Invitrogen) for concentration prior to sequencing on a MiSeq sequencer (Illumina) according to manufacturer’s instructions.

### Microbiota analysis

Analysis of the V4 region of the 16S rRNA gene was performed using the open-source software program mothur (v.1.41.1)^69^, according to the MiSeq standard operating procedure (http://www.mothur.org/wiki/MiSeq_SOP). The paired forward and reverse reads were assembled into contigs, and sequences longer than 275 base pairs, containing any ambiguous base calls, or a run of greater than 8 homopolymers were discarded. The remaining sequences were aligned to the Silva 16S rRNA reference files, release 132^70^. Chimeric sequences were identified and removed using the mothur implementation of the VSEARCH algorithm^71^. Reads were classified using the Ribosomal Database Project, version 9^72^, with a Bayesian classifier using an 80% bootstrap confidence level over 100 iterations. Contaminant sequences from mitochondria, chloroplasts, archaea, eukaryotes and unknown were removed. Remaining sequences were clustered into operational taxonomic units (OTUs), defined by a 97% similarity level, according to the average-neighbor algorithm. A total of 27,657 OTUs were identified. The samples contained an average of 207,798 sequences (range: 108,414–308,433). The percent relative abundance of bacterial family members in each sample was calculated. Samples were rarified to the lowest number of reads from a sample (108,414) to minimize the effects of different sequencing depths. The subsampled dataset contained 18,571 OTUs, with an average Good’s coverage of 99.0% (range: 98.4–99.2%). Alpha diversity was calculated by inverse Simpson index. Beta diversity was assessed using the Jaccard^63^, Bray-Curtis^64^, and Yue & Clayton theta (Θ_YC_)^65^ calculators. Visualization of the distance matrices was accomplished using principal coordinates analysis (PCoA).

The dataset we examined contained a large proportion of singletons (OTUs that contain a single sequence). To ensure that these unique events did not overly impact the data analysis, singletons were removed and the data were reanalyzed. Removal of the singletons decreased the number of unique OTUs in each group, but other measures of alpha-diversity (observed OTUs and inverse Simpson) were very similar to the dataset containing singletons. The beta-diversity of the samples was essentially identical, with PCoA graphs indistinguishable from the dataset with singletons (Data not shown). Statistical analysis of the distance matrices by analysis by molecular variance (AMOVA) reached the same significance. The only striking difference was the rarefaction curve; without singletons, the curves leveled off more than with singletons (though the curves still do not reach a slope of zero; data not shown). Overall, the removal of the singletons did not change the conclusions we made from the analysis, and OTU-based analysis was necessary to observe the differences between the groups by both beta-diversity and metastats. GraphPad Prism (8.0.0) was used to create heatmaps and to graph data. Quantitative Venn diagrams were based on those generated by BioVenn (http://www.biovenn.nl/index.php)^73^.

### Statistical analysis

Differences between treatment groups were tested by one-way ANOVA with Tukey’s test for multiple comparisons for the following comparisons: percent abundance of phylum-level sequences, observed OTUs, and inverse Simpson index. Significant differences in beta-diversity between the communities were determined with AMOVA using mothur. Differences in the abundance of specific OTUs between treatment groups were determined using the mothur implementation of Metastats^66^. For OTUs that were statistically different between groups, the sequences were additionally re-classified using the Greengenes reference files, version 13_8_99^74^.

## Supplementary information

Supplementary Information 1.

Supplementary Information 2.

Supplementary Information Table1.

Supplementary Information Table2.

Supplementary Information Table3.

## Data Availability

All the data supporting the results are included in the manuscript. The raw sequencing reads can be accessed using the National Center for Biotechnology Information (NCBI) BioProject ID# PRJNA561197 (http://www.ncbi.nlm.nih.gov/bioproject/561197). Samples M1 - M10 are the 10 SIV infected c-ART treated animals; samples M11 - M16 are the 6 SIV infected untreated animals; samples M18, M25, M27, and M29 - M32 are the 7 healthy animals.
