# Supplementary figures and images for: Microbial Dysbiosis During Simian Immunodeficiency Virus Infection is Partially Reverted with Combination Anti-retroviral Therapy
Source: Sci Rep. 2020 Apr 14;10:6387. doi: 10.1038/s41598-020-63196-0 (PMC7156522; doi:10.1038/s41598-020-63196-0)

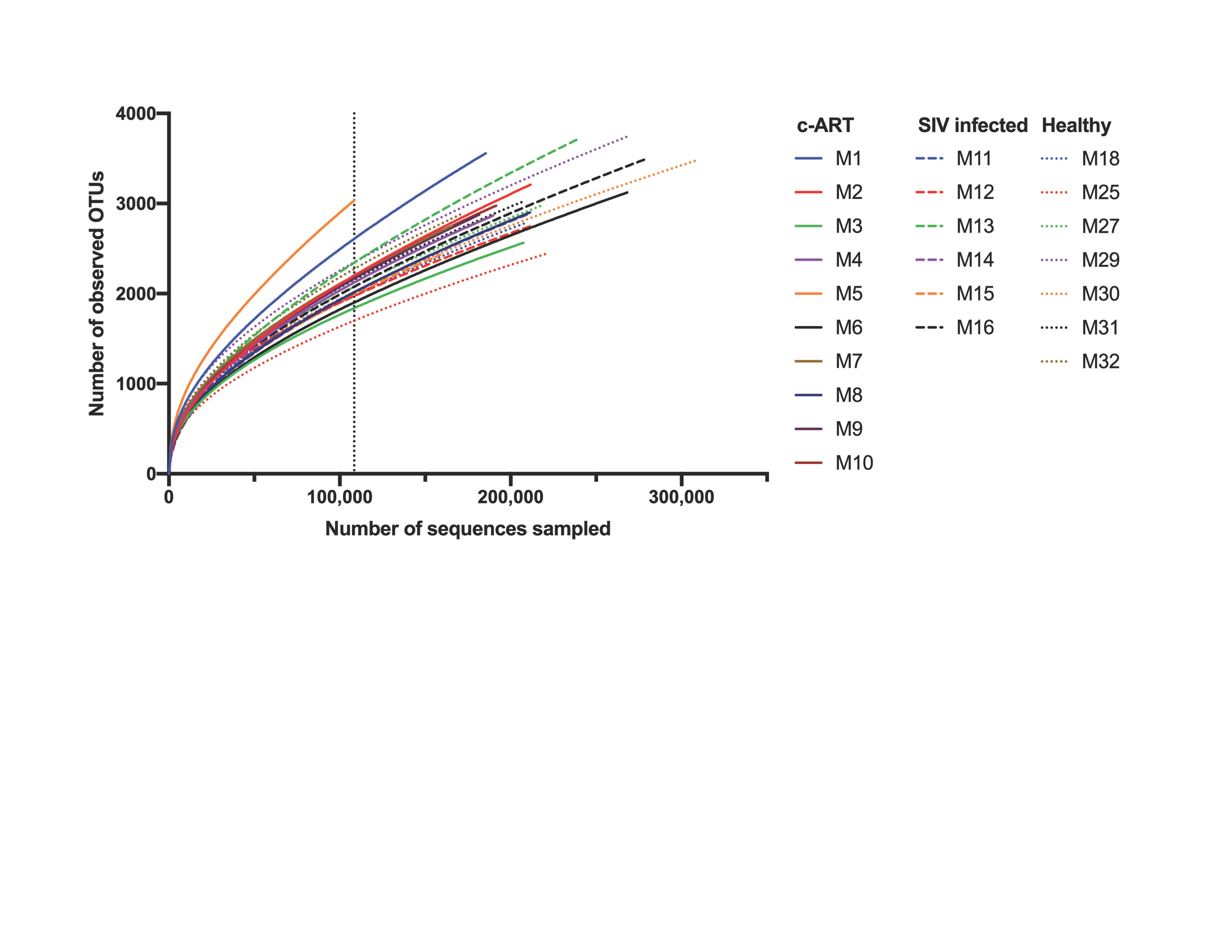

Supplement: Supplementary file 1 — Supplementary Information 1. [file 41598_2020_63196_MOESM1_ESM.jpg]

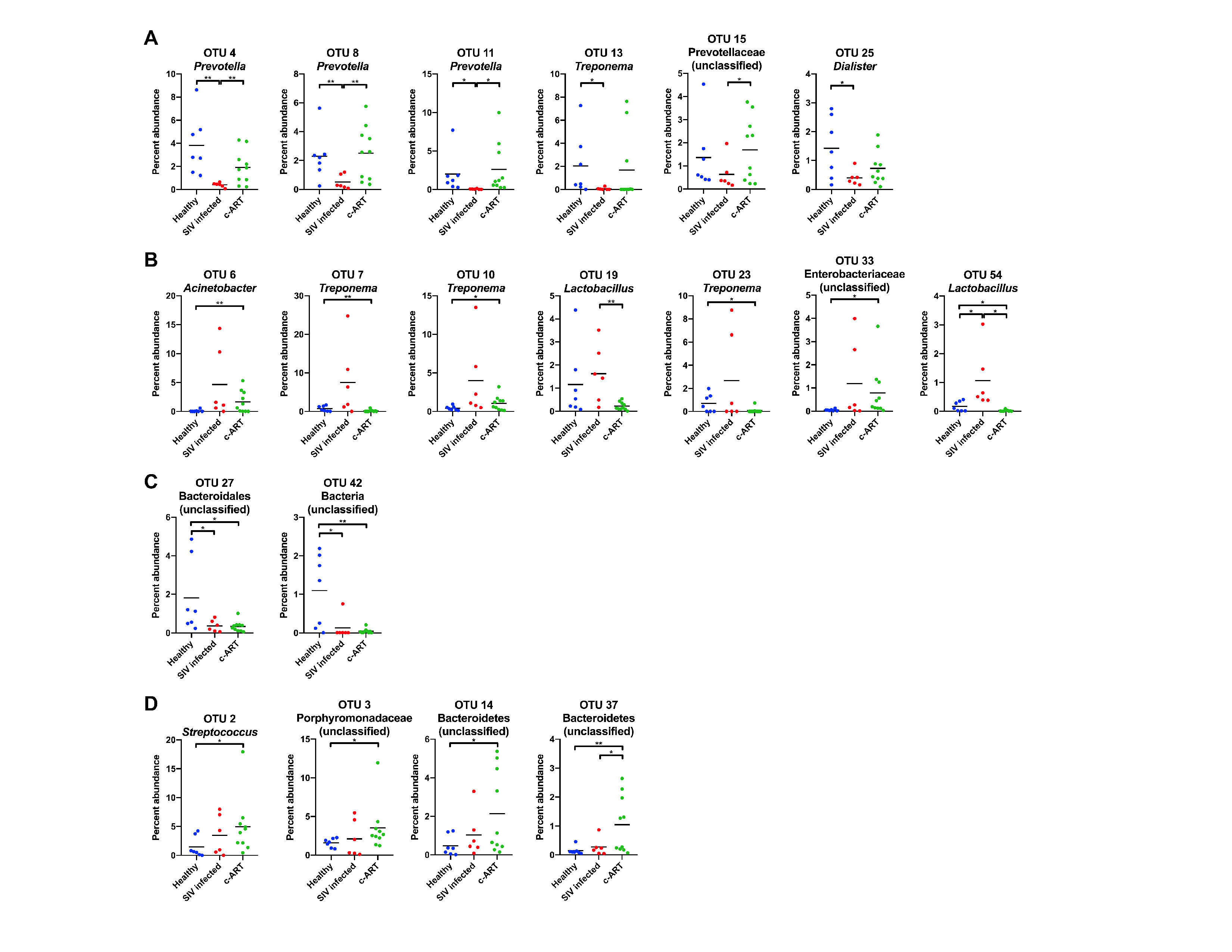

Supplement: Supplementary file 2 — Supplementary Information 2. [file 41598_2020_63196_MOESM2_ESM.jpg]
